# Relation Between Dietary Essential Fatty Acid Intake and Dry Eye Disease and Meibomian Gland Dysfunction in Postmenopausal Women

**DOI:** 10.1016/j.ajo.2018.01.004

**Published:** 2018-01-11

**Authors:** Jillian F. Ziemanski, Lynn R. Wolters, Lisa Jones-Jordan, Jason J. Nichols, Kelly K. Nichols

**Affiliations:** School of Optometry, University of Alabama at Birmingham, Birmingham, Alabama (J.F.Z., J.J.N., K.K.N.); and College of Optometry, The Ohio State University, Columbus, Ohio (L.R.W., L.J.-J.).

## Abstract

**PURPOSE:**

To evaluate the relationship between omega-3 (n-3) and omega-6 (n-6) fatty acids with dry eye disease (DED) and meibomian gland dysfunction (MGD).

**DESIGN:**

Cross-sectional study.

**METHODS:**

Postmenopausal women (n = 439) underwent a clinical evaluation and completed the Vio Food Frequency Questionnaire to estimate their dietary intake of n-3s and n-6s. Subjects were categorized into 2 binary classifications based on whether or not they had (1) DED and (2) MGD. Mean intake of dietary fatty acids was compared with 2-sample *t* tests. Univariate logistic regression models were used to estimate the odds ratios for each condition associated with each quintile of n-3s, n-6s, and n-6:n-3 ratios.

**RESULTS:**

For DED vs non-DED, there were no significant differences in n-3 intake (1.95 ± 1.47 g vs 1.92 ± 1.24 g, *P* = .86), n-6 intake (15.58 ± 11.56 g vs 15.44 ± 10.61 g, *P* = .91), and n-6:n-3 (8.30 ± 2.57 vs 8.30 ± 2.57, *P* = .99). For MGD vs non-MGD, there were no significant differences in n-3 intake (1.87 ± 1.35 vs 1.96 ± 1.39, *P* = .61), n-6 intake (15.26 ± 11.85 vs 15.62 ± 10.93, *P* = .80), and n-6:n-3 (8.35 ± 2.94 vs 8.28 ± 2.42, *P* = .84). The odds ratios (OR) for DED did not differ significantly from 1.0 for n-3, n-6, or n-6:n-3. High n-3 consumption (OR = 0.22 [0.06–0.78]) and moderate n-6 consumption (OR = 0.37 [0.15–0.91]) were associated with a decreased frequency of MGD.

**CONCLUSIONS:**

Dietary consumption of n-3s and n-6s showed no association with DED, but high n-3 consumption and moderate n-6 consumption were protective against MGD in this large sample of postmenopausal women.

Dry eye disease (ded) and meibomian gland dysfunction (MGD) are recognized as related entities that often have overlapping clinical presentations. Poor quality and/or reduced flow of meibum may result in a deficient tear film lipid layer and thus evaporative dry eye.^1^ In turn, chronic aqueous-deficient DED, possibly resulting in tear film hyperosmolarity and proinflammatory cytokines,^2^ may lead to damage to the meibomian gland ductal orifices and initiate MGD.^1^ Of the estimated 40 million individuals in the United States who struggle with dry eye, up to 78% of cases may be secondary to MGD.^3^ As reported in the 2011 Report of the International MGD Workshop, the prevalence of MGD varies from 3.5% to nearly 70%, with higher values observed in adult Asian populations.^4^ For both ocular surface conditions, postmenopausal women are believed to be one of the primary demographics most at risk, likely owing to hormonal dysregulation of the secretory glands.^4,5^

There is a growing body of literature aimed to investigate the effects of omega-3 (n-3) fatty acid supplementation as a treatment for ocular surface disease; inconclusive results are often reported, as reviewed previously.^6^ Importantly though, this direction of research stems from a 2005 report of the Women’s Health Study,^7,8^ stating that low dietary consumption, not supplementation, of omega-3s and high omega-6: omega-3 (n-6:n-3) ratios are associated with an increased incidence of self-reported dry eye disease.^9^ The mechanism has not been fully elucidated, but there is theoretical evidence in support of DED mitigation owing to n-3’s anti-inflammatory properties.^10^ Polyunsaturated fatty acids, such as n-3s and n-6s, yield prostaglandins and leukotrienes, both of which work through paracrine signaling to regulate inflammation and other physiologic processes.^10^ The n-6 precursors primarily give rise to proinflammatory eicosanoids, while the n-3 precursors primarily lead to anti-inflammatory eicosanoids. Therefore, diets rich in n-3s are believed to promote an anti-inflammatory physiologic state.^10^

The role of inflammation in MGD, however, is not clearly understood, as it does not appear to be ubiquitously present.^1^ Increased meibum viscosity, on the other hand, is part of the core pathophysiology of MGD.^1^ This, in conjunction with ductal keratinization, is believed to induce downstream obstructive and atrophic disease.^1^ Omega fatty acids are polyunsaturated fatty acids with multiple double bonds (existing in nature in the cis conformation) creating conspicuous kinks in their hydrocarbon chains.^11^ These kinks disrupt adjacent fatty acids from packing together tightly.^11^ Therefore, at any given temperature, polyunsaturated fatty acids are more fluid than saturated fatty acids. Diets high in polyunsaturated fatty acids, if these fatty acids are capable of being incorporated into the lipid macromolecules of meibum, could potentially improve meibum flow by decreasing its viscosity, thereby reducing ductal obstruction, a core mechanism of MGD.

Although the study by Miljanovic and associates^9^ was a pivotal investigation in nutrition and ocular surface disease, it was not designed to evaluate MGD, to confirm the presence of MGD or DED with a clinical examination, or to specifically recruit postmenopausal women. Therefore, the purpose of this analysis is to assess whether dietary consumption of n-3 and n-6 fatty acids, as well as the ratio between the two, is associated with an altered frequency of clinically confirmed DED or MGD in a large sample of postmenopausal women.

## METHODS

A single-center, cross-sectional study was conducted in an academic setting to assess differences between dry eye and normal ocular health in postmenopausal women, particularly in relation to the structure and function of the meibomian glands. The study was conducted in accordance with the tenets of the Declaration of Helsinki, and the study protocol was approved by the Institutional Review Board at The Ohio State University. All subjects provided informed consent, and confidentiality was maintained in full compliance with the HIPAA Privacy Rule.

### ENTRY CRITERIA AND STUDY DESIGN

Subjects were recruited from a multitude of sources: a clinical database of those interested in participating in clinical eye research, posted flyers, provider referrals, and word of mouth. To be eligible for the study, all subjects were required to pass the entry criteria during both a telephone screening and a subsequent clinical qualification visit. To ensure that only postmenopausal women were enrolled, only those who had at least 12 months of amenorrhea after age 50 were screened. The initial phone screening was employed to recruit subjects in an approximately 1:1 ratio to 1 of 2 groups: (1) potential dry eye disease or (2) normal controls of similar age range by decade. To be considered a potential dry eye subject, subjects had to report a previous diagnosis of dry eye disease or provide a response of “constantly” or “often” to the 2 symptom-based questions on the survey validated by Schaumberg and associates.^12^ Further, dry eye subjects were excluded if they were currently taking any prescribed ocular medication, including Restasis; had undergone any ocular surgery in the previous 12 months (except for yttrium-aluminum-garnet capsulotomy); had abnormal lid anatomy or position; were previously diagnosed with any other anterior segment disease, including, but not limited to, ocular allergy, significant anterior blepharitis, corneal infection, pterygia, pingueculitis, or any corneal dystrophy; or had any changes in hormone replacement therapy in the prior month. To be considered a normal control, all subjects had to deny a previous diagnosis of dry eye disease and provide a response of “never” or “rarely” to the 2 symptom-based questions on the survey by Schaumberg and associates: (1) “How often do your eyes feel dry?” and (2) “How often do your eyes feel irritated?”^12^ Further, all normal controls were required to meet the same exclusion criteria as detailed above. Individuals who passed the phone screening were then scheduled for a clinical qualification visit.

The examination consisted of a battery of validated questionnaires and several clinical dry eye tests. Subjects completed the Ocular Surface Disease Index (OSDI; Allergan, Inc, Irvine, California, USA)^13^ and the Vio Food Frequency Questionnaire (VioFFQ; VioCare, Inc, Princeton, New Jersey, USA).^14^ The clinical examination consisted of, in the sequence of testing, tear collection from the right eye for osmolarity (TearLab Osmolarity System; TearLab Corp, San Diego, California, USA), a comprehensive ocular surface and meibomian gland evaluation by slit-lamp biomicroscopy, fluorescein tear break-up time (TBUT), fluorescein corneal staining with Wratten #12 filter (NEI Dry Eye Workshop^15^), lissamine green conjunctival staining (NEI Dry Eye Workshop scale^15^), and Schirmer I test. The central 10 meibomian glands of the lower lids were examined for expressibility with a scale minimally adapted from the Report of the Diagnosis Subcommittee of the MGD Workshop (0 = normal, 1 = cloudy, 2 = granular, 3 = inspissated, 4 = no secretion).^16^

### SUBJECT CLASSIFICATION

Based on the results from the examination, all subjects were included in 2 analyses based on the following dichotomous classifications: (1) DED or non-DED and (2)MGD or non-MGD. The DED classification bore no influence on the MGD classification, and vice versa. DED was defined as meeting at least 2 of the following criteria: responses of “constantly” or “often” to both questions of the Schaumberg questionnaire, TearLab Osmolarity ≥ 308 mOsm/L for the right eye (the only eye sampled), sum corneal staining of both eyes ≥ 8 (sum of 5 corneal regions, graded on a 0–3 scale), average fluorescein TBUT < 7 seconds^17^ (3 consecutive measurements from both eyes), or average Schirmer ≤ 10 mm^17^ in 5 minutes (from both eyes). Those not meeting at least 2 of these criteria were categorized as non-DED. For the second analysis, MGD was defined as having at least granular meibum with digital pressure (average quality of both eyes ≥ 2 on a 0–4 scale). Those not meeting this criterion were categorized as non-MGD.

### DETERMINATIONOF FATTY-ACIDINTAKE

The VioFFQ is a validated, computer-based tool that collects information on dietary patterns and behavior to provide an estimate of nutrient intake. The questionnaire has since been renamed VioScreen, though its original name is used in this manuscript to avoid anachronistic terminology. From this questionnaire, mean dietary intake of n-3 and n-6 fatty acids, and the resultant n-6:n-3 ratio, were assessed so as to predict their relationship to DED/non-DED and MGD/non-MGD. The system was developed based on the National Institutes of Health–funded Women’s Health Initiative, one of the largest clinical trials of postmenopausal women’s health to date in the United States,^18^ and is therefore particularly sensitive to fat intake.^19^ The VioFFQ queries the frequency of dietary consumption over the previous 90 days for a multitude of common food items. The Nutrition Coordinating Center of the University of Minnesota, Division of Epidemiology and Community Health, maintains the Food and Nutrient Database that is used to generate estimates of nutrient intake in the VioFFQ. All de-identified FFQ data from all subjects were securely exported for external analysis and omega fatty acid nutrient estimation. The VioFFQ, now VioScreen, has recently been evaluated and is considered to be a reliable, accurate, and valid tool for collecting nutritional data.^20^ Additional information on this tool can be located on the manufacturer’s website (www.viocare.com) or in the reference by Kristal and associates.^20^

### STATISTICAL ANALYSES

All statistical calculations were performed using SAS v9.3 (SAS Institute, Cary, North Carolina, USA) by 1 author (L.J.J.). All clinical variables are summarized by the mean and standard deviation. Mean dietary intakes of n-3 fatty acids and n-6 fatty acids, and the resultant n-6:n-3 fatty acid ratios, were each compared between classifications (DED vs non-DED and MGD vs non-MGD) with 2-sample *t* tests. Similar to the methodology by Miljanovic and associates,^9^ the distributions of total n-3 consumption, total n-6 consumption, and n-6:n-3 ratios for all subjects were divided into quintiles, and differences across quintiles by disease status were assessed. The distribution of the n-6:n-3 ratio was also partitioned into the same 4 categories as described by Miljanovic and associates: <4:1, ≥4:1 to <10:1, ≥10:1 to <15:1, and ≥15:1. Two-sample *t* tests were used to assess for differences in clinical parameters, n-3 intake, n-6 intake, and n-6:n-3 intake ratios between DED/non-DED and MGD/non-MGD. One-way analysis of variance was used to assess for differences in n-3 intake, n-6 intake, and n-6:n-3 intake ratios across n-3 quintiles, n-6 quintiles, and n-6:n-3 quintiles. The χ2 test was used to assess for differences in DED frequency and MGD frequency across quintiles.

Univariate logistic regression models (multiple regression) were used to estimate the odds ratios for DED vs non-DED and MGD vs non-MGD across all quintiles of total n-3 intake, total n-6 intake, and n-6:n-3 intake ratios. The first model controlled for the following covariates: age, race, body mass index (BMI), total dietary fat intake, and presence or absence of an eye examination in the previous 12 months. A second model was also used to control for systemic comorbidities (hypertension, diabetes mellitus, and arthritis) in addition to the covariates from the initial model. Both of these models were applied to both binary classifications.

## RESULTS

**FIGURE 1:**
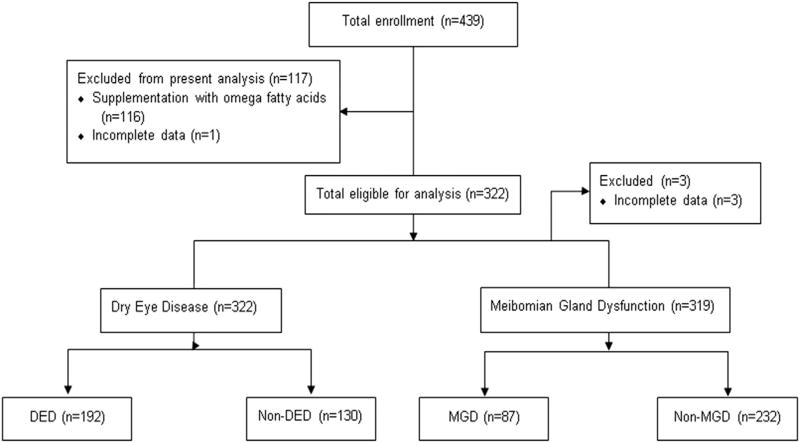
Enrollment flow chart. A total of 439 postmenopausal women were enrolled in the Dry Eye in Menopause study, but 117 were excluded from this analysis owing to baseline omega fatty acid supplementation (n = 116) or incomplete data (n = 1). Of the 322 women in the DED/non-DED analysis, 192 met the criteria to be diagnosed with DED, while 130 did not. Of the same 322 women in the MGD/non-MGD analysis, 3 were excluded owing to incomplete data. Of the 319 remaining, 87 met the criteria to be diagnosed with MGD, while 232 did not. DED = dry eye disease; MGD = meibomian gland dysfunction.

Four hundred thirty-nine subjects were enrolled in the Dry Eye in Menopause study (Figure 1). For this study, 116 individuals (26.4%) were excluded owing to baseline supplementation with any omega fatty acid supplements and 1 individual (0.2%) had incomplete supplement data, leaving 322 (73.3%) subjects eligible for the statistical analysis. Of these 322 subjects, 192 (59.6%) were categorized as meeting the definition for DED, while 130 (40.4%) were categorized as non-DED. For the MGD component, 3 of these same 322 subjects (0.9%) had incomplete data, leaving 319 (99.1%) available for analysis. Of these, 87 (27.3%) were classified as having MGD, while 232 (72.7%) were classified as non-MGD.

Clinical data for both classifications are displayed in Table 1. The DED group demonstrated excellent stratification by significantly differing from the non-DED group on nearly every parameter, even though only 2 parameters were required to have DED: dryness frequency, irritation frequency, corneal staining, osmolarity, TBUT, and Schirmer (all *P* < .0001). Only meibum quality, as expected, did not reach statistical significance between the 2 groups (*P* = .48). For the MGD classification, however, only TBUT (*P* = .003) was statistically significant. All other clinical parameters showed no significant differences from the non-MGD group, which is consistent with expected values for at least stage 1 MGD, as discussed later.

Across all subjects, the range of n-3 consumption was 0.06–11.08 g per day, and the range of n-6 consumption was 0.42–87.86 g per day. The ratio of n-6:n-3 ranged from 3.31 to 21.45. Table 2 displays the mean daily intake of n-3s and n-6s and n-6:n-3 ratios for each classification. There were no statistically significant differences in n-3 intake, n-6 intake, or n-6:n-3 ratios for those with DED compared to those without DED. Similarly, there were no statistically significant differences in n-3 intake, n-6 intake, or the n-6:n-3 ratios for those with MGD compared to those without MGD.

To determine whether increased consumption of omega fatty acids alters the frequency of DED or MGD, the distributions of total n-3 intake, total n-6 intake, and n-6:n-3 ratios were divided into quintiles, and the frequencies of DED and MGD were assessed between them (Table 3). There were no differences in the frequencies of DED or MGD across all five quintiles of n-3 intake, n-6 intake, or n-6:n-3 ratios. The frequencies of DED andMGD remained similar, regardless of the amount or type of omega fatty acids consumed in the diet. With both n-3s and n-6s, as well as the n-6:n-3 ratio, there were significant differences across age among the 5 quintiles, such that younger subjects tended to have higher amounts of n-3s and n-6s or higher n-6:n3 ratios (*P* = .002, *P* = .003, and *P* = .04, respectively). The mean age difference was 6.2 years between those in quintile 1 and those in quintile 5, as observed in the n-3 analysis. Though statistically significant, this difference is approximately 10% of the average age of the subjects and likely does not represent a physiologically meaningful or clinically relevant difference.

Assessment of the relative amounts of omega fatty acid intake revealed interesting consumption patterns (Table 2). As n-3 consumption increased from quintile 1 through quintile 5, n-6 intake also demonstrated an increase (*P* < .0001); however, the n-6:n-3 ratios decreased (*P* < .0001), suggesting that the increased consumption of both n-3s and n-6s was not proportional. In other words, the subjects in higher quintiles were likely consuming more foods rich in n-3s than in n-6s, allowing their n-6:n-3 ratio to be significantly smaller. Similarly in the n-6 analysis, there was also a nonproportional increase in both n-6 and n-3 consumption across the 5 quintiles (*P* < .0001), manifesting as a significant increase in the n-6:n-3 ratio (*P* < .0001). Subjects in higher quintiles were likely consuming more foods rich in n-6s than n-3s. In the n-6:n-3 ratio analysis, the rise in the ratios across the 5 quintiles is driven primarily by the rise in n-6 consumption (*P* < .0001), as there was no significant difference in n-3 consumption across the quintiles (*P* = .09). These results suggest that individuals consuming high ratios of n-6:n-3 fatty acids are doing so because of a disproportionately high amount of n-6 intake, not necessarily because of a deficiency in n-3 consumption.

**FIGURE 2:**
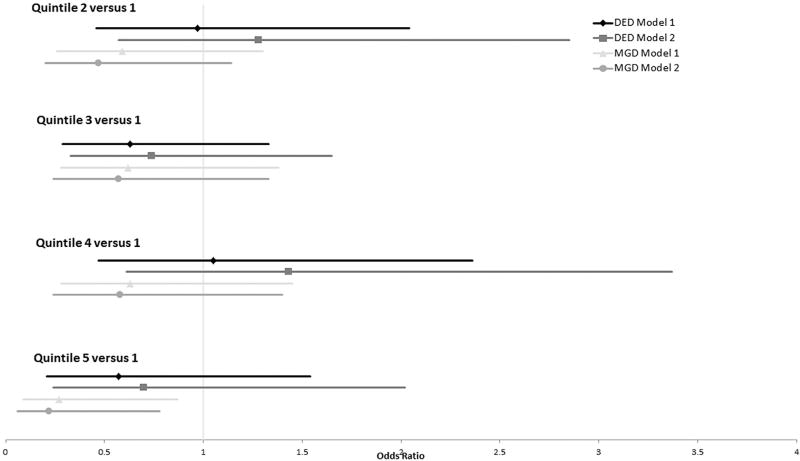
Odds ratio plot of dry eye disease and meibomian gland dysfunction with respect to omega-3 fatty acid consumption in postmenopausal women. Model 1 controlled for age, race, body mass index, total dietary fat intake, and presence or absence of an eye examination in the previous 12 months. Model 2 controlled for systemic hypertension, diabetes mellitus, and arthritis, in addition to the variables controlled for in Model 1. DED = dry eye disease; MGD = meibomian gland dysfunction.

**FIGURE 3:**
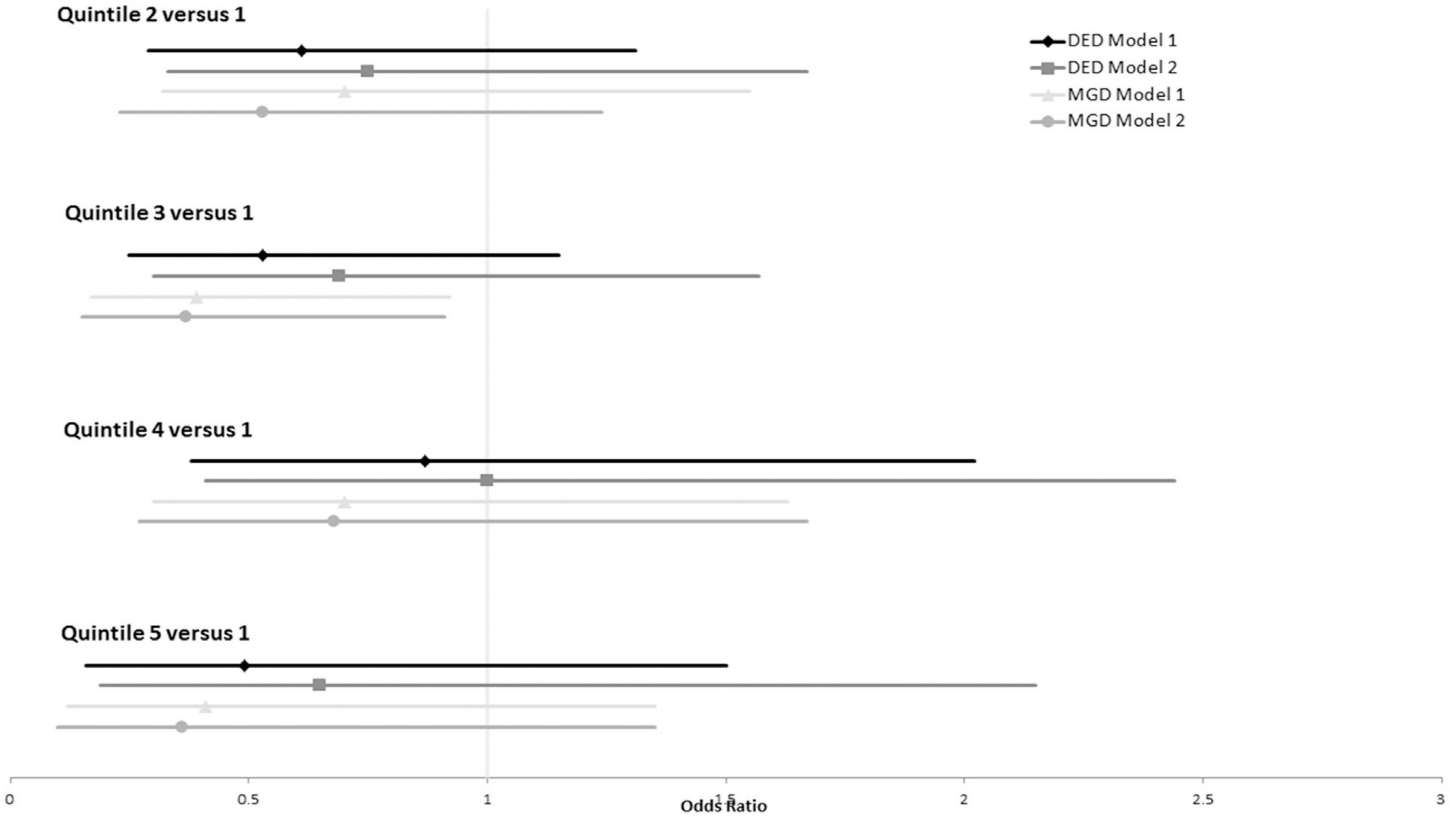
Odds ratio plot of dry eye disease and meibomian gland dysfunction with respect to omega-6 fatty acid consumption in postmenopausal women. Model 1 controlled for age, race, body mass index, total dietary fat intake, and presence or absence of an eye examination in the previous 12 months. Model 2 controlled for systemic hypertension, diabetes mellitus, and arthritis, in addition to the variables controlled for in Model 1. DED = dry eye disease; MGD = meibomian gland dysfunction.

**FIGURE 4:**
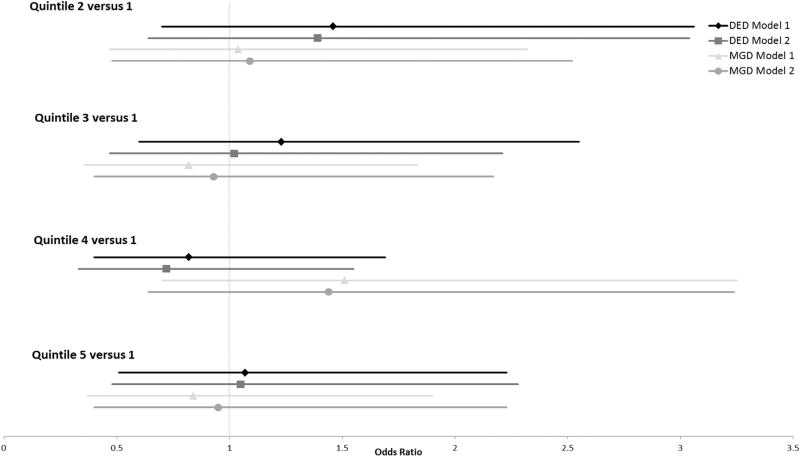
Odds ratio plot of dry eye disease and meibomian gland dysfunction with respect to omega-6:omega-3 fatty acid consumption in postmenopausal women. Model 1 controlled for age, race, body mass index, total dietary fat intake, and presence or absence of an eye examination in the previous 12 months. Model 2 controlled for systemic hypertension, diabetes mellitus, and arthritis, in addition to the variables controlled for in Model 1. DED = dry eye disease; MGD = meibomian gland dysfunction.

Univariate logistic regression models were used to estimate the odds ratio (OR) for each quintile and each ocular surface condition with respect to n-3 intake, n-6 intake, and n-6:n-3 ratio (Figures 2–4). After adjusting for all variables, the ORs for DED did not differ significantly from 1.0 across all 5 quintiles for n-3 intake, n-6 intake, or n-6:n-3 ratios. Those with the highest level of n-3 consumption (between 2.54 and 11.08 g) demonstrated a reduced frequency of MGD with both models (OR = 0.27 [0.09–0.87] and OR = 0.22 [0.06–0.78], respectively). No other n-3 quintile was associated with an increased or decreased frequency of MGD. For n-6 consumption, those in quintile 3 (11.33–14.56 g) demonstrated a reduced frequency of MGD with both models (OR = 0.39 [0.17–0.92] and OR = 0.37 [0.15–0.91], respectively). No other n-6 or n-6:n-3 quintile was associated with an increased or decreased risk of MGD.

The n-6:n-3 ratios were also divided into 4 pre-established ranges: <4:1, ≥4:1 to <10:1, ≥10:1 to <15:1, and ≥15:1.^9^ The odds ratios did not differ significantly from 1.0 for DED or MGD across all ranges, regardless of the logistic regression model used (Table 4).

## DISCUSSION

The aim of this analysis was to evaluate whether high n-3 and low n-6:n-3 dietary consumption are protective against DED and MGD in postmenopausal women. In this sample of 320 subjects, there was no observed increased or decreased frequency of DED relative to dietary intake of omega fatty acids, even among those with n-3 consumption, n-6 consumption, or n-6:n-3 ratios corresponding to high or low extremes. For MGD, however, high n-3 consumption and moderate n-6 consumption were associated with a decreased frequency of disease. There were no detected relations between n-6:n-3 ratios and MGD. Based on these results, n-3, n-6, and n-6:n-3 dietary consumption appear not to have an association with DED status in postmenopausal women, although dietary omega fatty acid consumption does seem to have some association with MGD status. This study did not assess the therapeutic value of n-3 supplementation in patients with these ocular surface conditions.

These findings are interesting given a previous research report^9^ and our current understanding of the immunologic roles of n-3 and n-6 fatty acids. It is known that these 2 families of polyunsaturated fatty acids are inconvertible and distinct in their chemical structures, yet each of their respective intermediates competes for the same enzymes, resulting in products that often have antagonistic functions.^10^ Downstream n-3 products lead to an eicosanoid profile that is primarily anti-inflammatory, while downstream n-6 products often shift toward a proinflammatory milieu.^10^ Because of their shared enzymatic pathways, diets rich in n-6s tend to promote more inflammation, a core process in DED, and diets rich in n-3s tend to suppress inflammation.^10^ Therefore, it might have been expected that an increased frequency of DED would have been observed at high levels of n-6 intake, while a decreased frequency of DED would have been observed at both high levels of n-3 intake and low ratios of n-6:n-3. Instead, no association was detected between n-3 and n-6 intake with respect to DED status.

The protective role of omega fatty acids in MGD specifically is largely understudied, possibly because the involvement of inflammation in the pathophysiology of MGD is considered sporadic.^1^ In theory, however, decreased saturation of these fatty acid moieties, which could potentially be provided by these polyunsaturated fatty acids, could lower the melting point of meibum, thereby increasing its fluidity at normal physiologic temperature. This theory, though, has not yet been supported by the literature, to our knowledge, and would suggest no preferential response to n-3s or n-6s, as they both are polyunsaturated fatty acids. In this study, we found a lower odds ratio for MGD with high n-3 consumption, possibly related to both quelling of any inflammation and increasing the flow of meibum. We also found that a defined range of n-6 consumption (11.33–14.56 g per day) was associated with a decreased frequency of MGD. It is possible that an ideal range of n-6 consumption exists; however, it is also possible that the overall fewer number of MGD subjects (n = 87) compared to non-MGD subjects (n = 232) is contributing to a type I error. Further research is needed to better elucidate these relationships and to more definitively investigate the underlying mechanism linking omega fatty acids to MGD specifically.

The prevalence of MGD in population-based studies has been reported to vary between 3.5% and 69.3%, with lower values corresponding to white populations and higher values to Asian populations.^21–26^ In a sample of 398 participants from California, 38.9% were reported to have cloudy or absent secretion of meibum from their lower lids.^27^ In our analysis, we defined MGD to be a clinically significant reduction in meibum quality with a grade of 2 on a 0–4 scale. Of the 319 participants in the MGD/non-MGD analysis, 87 (27.3%) met the criterion for MGD. Our participants were recruited from central Ohio, predominantly white, and exclusively postmenopausal, providing reasonable concordance with previously published values of MGD frequency and/or prevalence. Variability in disease definitions has plagued both MGD and DED epidemiologic literature. For this study, a simple diagnostic algorithm that was consistent with the MGD Workshop of 2011 was most appropriate. Any subject who met the criteria for stage 1 MGD (no symptoms, clinical signs based on gland expression, and no ocular surface staining), as outlined in the Executive Summary,^28^ were considered to have MGD. As stated in the Report of the Diagnosis Subcommittee,^16^ gland expression is largely based on meibum quality using a scale comparable to the one that was used in this study. Per this report, a score of 1 is acceptable as normal, but a score greater than 1 is abnormal. Therefore, we used a cut point of ≥2 to be consistent with the Report of the MGD Workshop. While a more relaxed definition of MGD (eg, meibum quality ≥ 1) would have resulted in a much higher frequency, our aim was to focus on clinical significance corresponding to a grade of 2 or higher.

These analyses failed to replicate the previous 2005 findings of Miljanovic and associates from the Women’s Health Study,^9^ who found that women with higher consumption of n-3 fatty acids had a decreased risk of DED, while women with a high n-6:n-3 ratio had a greater risk of DED. It is important to recall that DED was not clinically confirmed in the Women’s Health Study, which could present one possibility for our disparate results. Additionally, the Women’s Health Initiative was a longitudinal investigation, allowing the authors to assess the incidence of DED over a 4-year period. The present study was a cross-sectional investigation, thereby assessing the frequency of DED and MGD among postmenopausal women. Regardless of these differences, our results are in agreement with Galor and associates.^29^ They recently reported no benefit of a diet high in n-3 fatty acids with regard to clinically confirmed DED in a sample of 247 men between the ages of 55 and 95. To the authors’ knowledge, these 3 reports are the only known reports assessing omega fatty acid intake by a computer-based food frequency questionnaire with respect to DED and MGD.

Of note, there are 2 significant differences in the study designs of these 3 reports: sex profile (all female vs all male) and menopausal status (no specification vs solely postmenopausal).^9,29^ Lipid metabolism is known to vary widely between the sexes and between pre- and post-menopause.^30^ To date, investigation of lipid metabolism within the meibomian gland in response to parameters such as diet content, temporality to meals (postprandial vs preprandial), abdominal obesity, sex, menopausal status, etc, is significantly lacking.

Though treatment is not the specific topic of this investigation, we would be remiss not to acknowledge the recent proliferation of clinical trials assessing the efficacy of omega fatty acids in dry eye disease. Since 2005, the year that the dry eye data from the Women’s Health Study were published, there have been at least 20 clinical trials assessing omega fatty acids in dry eye and dry eye–related conditions. A review of these trials through 2015 has previously been published^6^; further review is beyond the scope of this manuscript. Of note, however, is that there are significant inter-experimental differences in the study designs, such as in subject classifications, drug formulation, treatment periods, and outcomes, complicating the ability to replicate findings and draw strong conclusions. The most prominent difference is a lack of a standard omega-3 and omega-6 composition for the interventional product, as well as lack of a standard and inert placebo. Studies have used products with primarily omega-3s, primarily omega-6s, and differing ratios of omega-6:omega-3. Similarly, placebos have been equally varied: fructose,^31^ olive oil,^32,33^ medium-chain triglycerides,^34–36^ wheat germ oil,^37^ sunflower oil,^38,39^ and corn oil.^40^ All of these oil products consist of some ratio of omega-3, -6, and/or -9 fatty acids, and, therefore, are accompanied by at least some biological activity. Without consistency in study design and placebo products, results are variable.

Despite these key differences, there does seem to be a trend toward improved DED or MGD status across many of the trials, though the parameters yielding improvement are quite variable. Beyond signs and symptoms, omega fatty acid supplementation has been shown to increase tear levels of prostaglandin E1,^31^ reduce HLA-DR^35,38^ and CD11^38^ surface expression, and decrease tear concentrations of interleukin (IL)-1β, IL-6, and IL-10,^41^ all of which support an anti-inflammatory mechanism at the ocular surface. Largely, however, many of these trials fail to acknowledge omega fatty acid bioavailability,^42^ which can vary between omega-3 and -6 formulations and can be estimated with erythrocyte fatty acid analysis. For example, recently, Epitropoulos and associates reported improvement in tear osmolarity, TBUT, MMP-9, and OSDI scores with a re-esterified fish oil formulation, which is purportedly more bioavailable.^43^ Deinema and associates evaluated differences between the phospholipid form (krill oil) and triacylglyceride form (fish oil).^44^ Although both mitigated disease relative to placebo, the phospholipid form (krill oil) may have additional benefit on OSDI scores and IL-17A tear levels. By incorporating relevant fatty acid indices to assess bioavailability, all clinical findings could be compared to the percentage of omega-3s (or -6s) present in the erythrocyte cell membranes, thereby controlling for many of the variabilities in study design. Importantly, our study also did not evaluate erythrocyte fatty acids, as our primary objective was to assess dietary consumption of omega fatty acids in postmenopausal women in a design similar to that of Miljanovic and associates.^9^ Future studies, however, should incorporate this marker to better understand the effect of bioavailability.

To better understand the underlying mechanism and to control for confounding variables between subjects, some researchers have investigated similar questions, but in a preclinical, cellular model. An immortalized human meibomian gland epithelial cell (HMGEC) line was previously developed.^45^ Hampel and associates^46^ found that HMGECs treated with docosahexaenoic acid, an n-3 fatty acid, upregulated lipid production, while human corneal epithelial cells demonstrated no lipid accumulation. In another study, Liu and associates^47^ supplemented the same cell line with n-3s, n-6s, and a combination of both and also found an increase in neutral lipid production, the primary lipid component of human meibum. These results suggest that n-3 and n-6 fatty acids work directly on the meibomian glands to increase lipid production. Despite this evidence, these findings do not seem to translate into relevant clinical findings observed in our cohort of postmenopausal women. One possibility is owing to the inherent and extensive inter-individual variability that complicates all clinical research. Another possibility is that the HMGEC line originated from a male donor, and as previously mentioned, lipid metabolism is known to differ between the sexes.^30^

It is important to acknowledge the limitations of this investigation. We implemented a cross-sectional study design in a disease associated with significant temporal variability.^48,49^ Secondly, any questionnaire that surveys an individual’s recollection of his or her dietary patterns is subject to possible error owing to intentional or unintentional misreporting on behalf of the subject or incomplete assessment on behalf of the survey developers. Because of this inherent possibility for error, we used the VioFFQ,^20^ a questionnaire developed based on the Women’s Health Initiative FFQ that was developed to be especially sensitive to dietary fat intake.^19^ Lastly, tear osmolarity was measured only once from the right eye in this study. Data acquisition occurred prior to discovering the value of repeated measurements^50^ and of bilateral testing^51^ in osmolarity. Future investigations should consider incorporating these aspects into their study designs and statistical analyses.

In conclusion, our systematic investigation of risk profiles stratified by omega fatty acid intake in 322 postmenopausal women provides further evidence that the role of n-3 dietary consumption alone as a protective factor for DED is dubious. Additional research is needed to further replicate our results and to determine whether n-3 supplementation or modulation of n-6 intake is of greater benefit in MGD specifically.

## Figures and Tables

**TABLE 1:** Clinical Data for Each Classification

Parameter	Non-DED (N = 130)	DED (N = 192)	*t* Statistic (*P* Value)	Non-MGD (N = 232)	MGD (N = 87)	*t* Statistic (*P* Value)
Dryness frequency	1.77 ± 0.78	2.54 ± 0.95	−7.95 (*P* < .0001*)	2.19 ± 0.92	2.32 ± 1.06	−1.13 (*P* < .26)
Irritation frequency	1.88 ± 0.51	2.46 ± 0.76	−8.13 (*P* < .0001*)	2.20 ± 0.71	2.29 ± 0.75	−0.98 (*P* < .33)
Corneal staining	2.55 ± 3.04	6.97 ± 5.62	−9.09 (*P* < .0001*)	4.81 ± 4.74	6.26 ± 6.28	−1.97 (*P* < .051)
Osmolarity (mOsm/L)	299.9 ± 14.3	310.3 ± 21.2	−5.18 (*P* < .0001*)	306.4 ± 18.9	305.7 ± 21.0	0.26 (*P* < .79)
TBUT (s)	11.10 ± 6.05	5.51 ± 3.78	−9.33 (*P* < .0001*)	8.28 ± 5.78	6.41 ± 4.69	2.98 (*P* < .003*)
Schirmer (mm/5 min)	18.68 ± 9.50	10.96 ± 8.30	7.67 (*P* < .0001*)	13.61 ± 9.21	15.15 ± 10.50	−1.27 (*P* < .20)
Meibum quality	1.12 ± 1.12	1.21 ± 1.08	−0.70 (*P* = .48)	0.61 ± 0.51	2.68 ± 0.74	−24.05 (*P* < .0001*)

DED = dry eye disease; MGD = meibomian gland dysfunction; TBUT = tear break-up time.

DED was defined as having at least 2 of the following criteria: responses of “constantly” or “often” to both questions of the Schaumberg questionnaire, osmolarity ≥ 308 for the right eye (the only eye sampled), sum corneal staining of both eyes ≥ 8, average fluorescein TBUT < 7 seconds, and average Schirmer ≤ 10 mm in 5 minutes. MGD was defined as having at least granular meibum with digital pressure (≥2).

All values are listed as mean ± standard deviation. All unitless values were graded on a scale of 0–4.

Asterisk (*) denotes statistically significant *P* value.

**TABLE 2:** Mean Daily Omega Fatty Acid Intake for Each Classification

	DED	Non-DED	*t* Statistic (*P* Value)	MGD	Non-MGD	*t* Statistic (*P* Value)
n-3 (g)	1.95 ± 1.47	1.92 ± 1.24	−0.17 (*P* = .86)	1.87 ± 1.35	1.96 ± 1.39	0.51 (*P* = .61)
n-6 (g)	15.56 ± 11.52	15.47 ± 10.66	−0.06 (*P* = .95)	15.24 ± 11.85	15.62 ± 10.98	0.26 (*P* = .79)
n-6:n-3	8.28 ± 2.57	8.33 ± 2.58	0.17 (*P* = .86)	8.35 ± 2.94	8.29 ± 2.43	−0.20 (*P* = .84)

DED = dry eye disease; MGD = meibomian gland dysfunction; n-3 = omega-3 fatty acids; n-6 = omega-6 fatty acids; n-6:n-3 = ratio of omega-6 fatty acids to omega-3 fatty acids.

All values are listed as mean ± standard deviation.

**TABLE 3:** Odds Ratios for Each Classification Divided Into Quintiles

	1	2	3	4	5	*P* for Trend
n-3						
Range (g)	0.06–1.05	1.05–1.44	1.44–1.88	1.90–2.53	2.54–11.08	
No. of subjects	64	64	65	64	65	
Mean age (years)	65.8 ± 10.2	63.0 ± 9.6	61.3 ± 7.4	61.8 ± 7.5	59.6 ± 8.6	.002
Mean n-3 intake (g)	0.73 ± 0.24	1.22 ± 0.11	1.66 ± 0.13	2.16 ± 0.18	3.89 ± 1.86	<.001*^a^
Mean n-6 intake (g)	6.65 ± 3.40	11.10 ± 3.62	13.12 ± 3.60	16.46 ± 4.60	30.09 ± 15.71	<.001*
Mean n-6:n-3	8.94 ± 2.83	9.17 ± 3.06	7.94 ± 2.22	7.64 ± 2.15	7.82 ± 2.13	<.001*
DED (%)	62.5	62.5	52.3	65.6	55.4	.50
Non-DED (%)	37.5	37.5	47.7	34.4	44.6	
DED OR Model 1 (95% CI)	1.0	0.97 (0.46–2.04)	0.63 (0.29–1.33)	1.05 (0.47–2.36)	0.57 (0.21–1.54)	.44
DED OR Model 2 (95% CI)	1.0	1.28 (0.57–2.85)	0.74 (0.33–1.65)	1.43 (0.61–3.37)	0.70 (0.24–2.02)	.31
MGD (%)	32.8	25.4	26.6	29.7	21.9	.69
Non-MGD (%)	67.2	74.6	73.4	70.3	78.1	
MGD OR Model 1 (95% CI)	1.0	0.59 (0.26–1.30)	0.62 (0.28–1.38)	0.63 (0.28–1.45)	0.27 (0.09–0.87)	.26
MGD OR Model 2 (95% CI)	1.0	0.47 (0.20–1.10)	0.57 (0.24–1.33)	0.58 (0.24–1.40)	0.22 (0.06–0.78)	.16
n-6						
Range (g)	0.42–8.25	8.49–11.32	11.33–14.56	14.57–20.21	20.27–87.85	
No. of subjects	64	64	65	64	65	
Mean age (years)	65.5 ± 10.1	62.8 ± 8.9	62.6 ± 8.9	61.3 ± 7.7	59.4 ± 7.9	.003*
Mean n-3 intake (g)	0.84 ± 0.41	1.34 ± 0.32	1.68 ± 0.41	2.15 ± 0.52	3.65 ± 2.04	<.001*
Mean n-6 intake (g)	5.63 ± 1.93	10.10 ± 0.82	12.89 ± 0.95	16.78 ± 1.75	32.00 ± 14.33	<.001*^a^
Mean n-6:n-3	7.32 ± 2.02	7.88 ± 1.75	8.15 ± 2.16	8.39 ± 2.89	9.73 ± 3.16	<.001*
DED (%)	64.1	57.8	53.8	65.6	56.9	.62
Non-DED (%)	35.9	42.2	46.2	34.4	43.1	
DED OR Model 1 (95% CI)	1.0	0.61 (0.29–1.31)	0.53 (0.25–1.15)	0.87 (0.38–2.02)	0.49 (0.16–1.50)	.38
DED OR Model 2 (95% CI)	1.0	0.75 (0.33–1.67)	0.69 (0.30–1.57)	1.00 (0.41–2.44)	0.65 (0.19–2.15)	.77
MGD (%)	33.3	28.1	18.5	30.6	26.1	.39
Non-MGD (%)	66.7	71.9	81.5	69.4	73.9	
MGD OR Model 1 (95% CI)	1.0	0.70 (0.32–1.55)	0.39 (0.17–0.92)	0.70 (0.30–1.63)	0.41 (0.12–1.35)	.22
MGD OR Model 2 (95% CI)	1.0	0.53 (0.23–1.24)	0.37 (0.15–0.91)	0.68 (0.27–1.67)	0.36 (0.10–1.35)	.22
n-6:n-3						
Range (g)	3.31–6.43	6.44–7.32	7.32–8.36	8.36–9.78	9.82–21.45	
No. of subjects	64	64	65	64	65	
Mean age (years)	64.4 ± 10.4	60.6 ± 7.9	60.6 ± 8.0	63.7 ± 8.7	62.3 ± 9.0	.04*
Mean n-3 intake (g)	2.22 ± 1.38	2.18 ± 1.79	1.83 ± 1.02	1.72 ± 1.19	1.74 ± 1.36	.09
Mean n-6 intake (g)	12.28 ± 7.20	15.00 ± 12.49	14.28 ± 7.64	15.36 ± 10.61	20.64 ± 14.57	<.001*
Mean n-6:n-3	5.54 ± 0.80	6.86 ± 0.24	7.84 ± 0.32	8.98 ± 0.40	12.23 ± 2.47	<.001*^a^
DED (%)	57.8	64.1	61.5	53.1	61.5	.75
Non-DED (%)	42.2	35.9	38.5	46.9	38.5	
DED OR Model 1 (95% CI)	1.0	1.46 (0.70–3.06)	1.23 (0.60–2.55)	0.82 (0.40–1.69)	1.07 (0.51–2.23)	.48
DED OR Model 2 (95% CI)	1.0	1.39 (0.64–3.04)	1.02 (0.47–2.21)	0.72 (0.33–1.55)	1.05 (0.48–2.28)	.60
MGD (%)	27.0	27.0	23.1	36.5	23.1	.42
Non-MGD (%)	73.0	73.0	76.9	63.5	76.9	
MGD OR Model 1 (95% CI)	1.0	1.04 (0.47–2.32)	0.82 (0.36–1.83)	1.51 (0.70–3.25)	0.84 (0.37–1.90)	.53
MGD OR Model 2 (95% CI)	1.0	1.09 (0.48–2.52)	0.93 (0.40–2.17)	1.44 (0.64–3.24)	0.95 (0.40–2.23)	.83

All means are expressed as mean ± standard deviation. Model 1 controlled for age, race, body mass index, total dietary fat intake, and presence or absence of an eye examination in the previous 12 months. Model 2 controlled for systemic hypertension, diabetes mellitus, and arthritis, in addition to the variables controlled for in Model 1.

CI = confidence interval; DED = dry eye disease; MGD = meibomian gland dysfunction; n-3 = omega-3 fatty acids; n-6 = omega-6 fatty acids; n-6:n-3 = ratio of omega-6 fatty acids to omega-3 fatty acids; OR = odds ratio.

Asterisk (*) denotes statistically significant *P* value.

a *P* values corresponding to data that were intentionally used to stratify participants across the quintiles.

**TABLE 4:** Odds Ratios for Each Preset Ratio Level of Omega-6 to Omega-3 Fatty Acid Consumption

	Ratio Level			
	
Model	<4:1	≥4:1 to <10:1	≥10:1 to <15:1	≥15:1
DED model 1	1.0	0.52 (0.05–5.34)	0.47 (0.04–5.23)	0.35 (0.03–6.25)
DED model 2	1.0	0.55 (0.05–6.11)	0.56 (0.05–6.76)	0.46 (0.02–8.50)
MGD model 1	1.0	0.41 (0.06–3.06)	0.24 (0.03–2.04)	1.09 (0.08–14.23)
MGD model 2	1.0	0.46 (0.06–3.50)	0.34 (0.04–2.98)	1.41 (0.10–19.16)

DED = dry eye disease; MGD = meibomian gland dysfunction.

All values are expressed as odds ratio ± 95% confidence interval.
